# A Comparison of the Inflammatory and Proteolytic Effects of Dung Biomass and Cigarette Smoke Exposure in the Lung

**DOI:** 10.1371/journal.pone.0052889

**Published:** 2012-12-20

**Authors:** Divya Mehra, Patrick M. Geraghty, Andrew A. Hardigan, Robert Foronjy

**Affiliations:** 1 Department of Medicine, Columbia University College of Physicians and Surgeons, New York, New York, United States of America; 2 Division of Pulmonary and Critical Care Medicine, St. Luke's Roosevelt Hospital Center, New York, New York, United States of America; University of Rochester Medical Center, United States of America

## Abstract

**Rationale:**

Biomass is the energy source for cooking and heating for billions of people worldwide. Despite their prevalent use and their potential impact on global health, the effects of these fuels on lung biology and function remain poorly understood.

**Methods:**

We exposed human small airway epithelial cells and C57BL/6 mice to dung biomass smoke or cigarette smoke to compare how these exposures impacted lung signaling and inflammatory and proteolytic responses that have been linked with disease pathogenesis.

**Results:**

The *in vitro* exposure and siRNA studies demonstrated that biomass and cigarette smoke activated ERK to up regulate IL-8 and MMP-1 expression in human airway epithelial cells. In contrast to cigarette smoke, biomass also activated p38 and JNK within these lung cells and lowered the expression of tissue inhibitor of matrix metalloproteinase-1 (TIMP-1). Similarly, in the lungs of mice, both biomass and cigarette smoke exposure increased macrophages, activated ERK and p38 and up regulated MMP-9 and MMP-12 expression. The main differences seen in the exposure studies was that mice exposed to biomass exhibited more perivascular inflammation and had higher G-CSF and GM-CSF lavage fluid levels than mice exposed identically to cigarette smoke.

**Conclusion:**

Biomass activates similar pathogenic processes seen in cigarette smoke exposure that are known to result in the disruption of lung structure. These findings provide biological evidence that public health interventions are needed to address the harm associated with the use of this fuel source.

## Introduction

In the developing world, it is estimated that air pollution from biomass smoke accounts for 2.2 to 2.5 million deaths annually [1]. Epidemiologic studies have implicated biomass use in the development of chronic obstructive pulmonary disease (COPD) in adults and acute lower respiratory infection in children [2], [3]. Women are particularly affected given their daily usage of these fuel sources for cooking. Moreover, exposure in women begins early in life and continues for decades [4]. Indeed, several studies have found increased markers of inflammation and oxidative stress in premenopausal women exposed to biomass smoke [5]–[7]. Worldwide, it is estimated that three billion people utilize biomass as their primary source of domestic energy [8]. Thus, understanding how biomass smoke affects lung biology and function is an important question that has significant public health implications.

In India, biomass accounts for approximately 90% of primary energy use broken down as wood in 56% of cases, dung in 21% and crop residues in 16% [9]. Despite its prevalence, the impact of biomass fuel on the health of exposed individuals remains poorly understood. Even less is known about the specific processes responsible for the ill effects of dung biomass. This study sought to better understand the biological consequences of dung biomass combustion on individuals utilizing the fuel for cooking. Through the development of both an *in vitr*o and *in vivo* model of biomass exposure, this work detailed the biological mechanisms by which this exposure mediates lung damage. Moreover, comparative analyses were conducted with cigarette smoke in order to determine whether biomass activated pathogenic mechanisms linked to the development of COPD.

## Materials and Methods

### Biomass and cigarette smoke extract preparation

**Figure 1:**
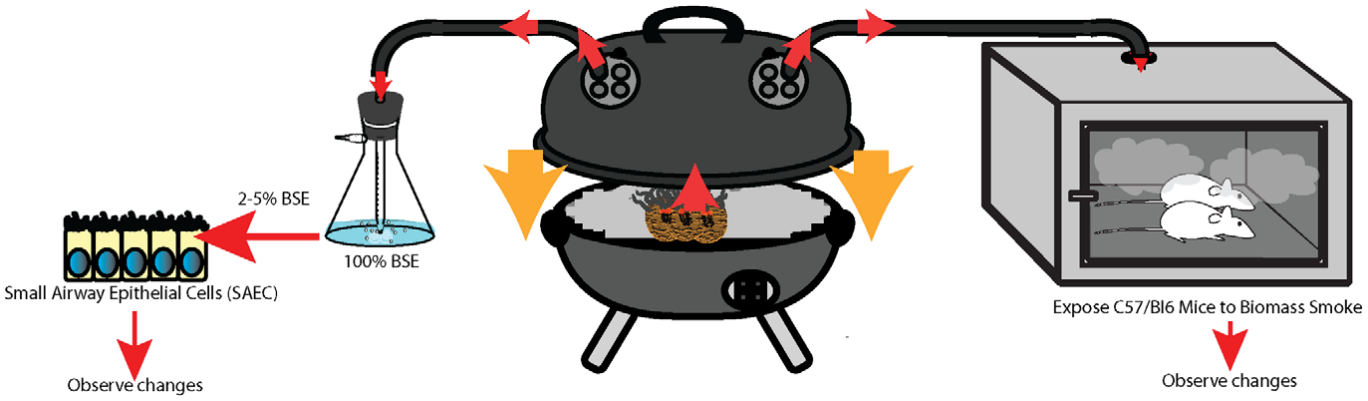
Schematic of the *in vitro* and *in vivo* exposure models. Dung biomass from the village studied in North India was combusted on a barbecue grill. The smoke was channeled to either create biomass smoke extract (BSE) for the *in vitro* studies or it was directed to the mouse chamber for *in vivo* exposure studies. The total particulate matter concentration within the chamber was maintained at 200 mg/m^3^ for the duration of the exposure.

Cigarette smoke extract was prepared by bubbling the smoke of one cigarette (3R4F, University of Kentucky, Lexington, KY) through 25 ml of PBS for ten minutes. The cigarette smoke extract was pH balanced to 7.4 and sterile filtered prior to use [10]. Dried biomass obtained from North India was burned in a barbecue grill and then channeled through PBS to prepare biomass smoke extract (BSE) (**Fig. 1**) using the same methodology to make cigarette smoke extract (CSE) [10]. Specifically, a dung biomass cake was burned on the grill for 9 minutes while the smoke produced was channeled through 25 ml of PBS. The PBS was then sterile filtered and pH balance to 7.4 prior to use. Endotoxin was removed from BSE as previously described [11]. Of note, the optical density measured at 320 and 595 nm was used to compare the concentration of BSE and CSE. Based on these measurements, the particulate matter content was comparable between the two forms of extract.

### *In vitro* studies of biomass and cigarette exposure

Human small airway epithelial cells (SAECs) (Lonza, Walkersville, MD) were cultured and Western blot analysis for MAPK signaling and ELISAs to detect the level of MMP-1, MMP-9, TIMP1 and IL-8 were performed as previously reported [10]. The cells were treated with 2–5% of BSE, 2–5% of CSE, 1 µg/ml LPS from *Escherichia coli* strain 0111:B4 or 50 or 100 μM of naphthalene, which was analyzed since it is a primary component of biomass smoke [12]. A 24-hour time point was chosen for ELISA analyses and a 30-minute time point was chosen for the Western analysis. SAE cell viability was tested using two different methods, with an Alamar Blue Reagent cell viability assay (Invitrogen), and by measurement of lactate dehydrogenase (LDH) release using a LDH cytotoxicity kit (Sigma Aldrich). To determine the specific signaling pathways responsible for the induction of lung cytokines and proteases, SAECs were grown to 50–60% confluence in 6-well plates and then each well was treated with 6 μl of Lipofectamine (Ambion, Grand Island, NY) transfection reagent containing 12.5 pmol of siRNA (Ambion, Grand Island, NY) for control siRNA, mitogen activated protein kinase 3 (MAPK3 or ERK), p38 MAP Kinase or mitogen activated protein kinase 8 (MAPK8 or JNK). After a six-hour incubation, the media was exchanged with fresh media or media containing 5% CSE or BSE for 24 hours. Multiplex analyses for IL-8, MMP-3 and -9 (BioRad, Hercules, CA) were conducted on the media collected from the treated cells. To confirm gene silencing, Western blot for ERK, p38 and JNK were also conducted on the cell lysate protein collected from these cells using specific rabbit polyclonal antibodies (Cell Signaling, Danvers, MA).

### *In vivo* studies of biomass exposure

For the mouse exposure studies, 8-week old C57BL/6 (Jackson Labs, ME) were exposed for 7 consecutive days to room air, cigarette smoke for 6 hours daily or dung smoke for 1 hour a day. The dung smoke was produced using the same experimental design as in our *in vitro* cigarette exposure system. Instead of passing the smoke through PBS as in the *in vitro* system, the smoke produced from the grill was let into a specially designed chamber containing the mice, and the total particulate matter concentration in the chamber was regulated at 200 mg/m^3^ (204±9 mg/m^3^; n = 10 measurements) **(** **Fig. 1** **)**. To approximate the experience of those cooking with dung biomass, we opted for a short but more intense exposure. Food and drinking water were provided ad libitum. Mice were exposed to cigarette smoke in a specially designed chamber (Teague Enterprises, Davis, CA, USA) for six hours a day at a total particulate matter concentration of 80 mg/m^3^ as per our established protocol [13]. N = 10 in each group. Lung lavage cellularity, Western blot analysis for MAPK signaling, histological analysis and quantitative PCR were conducted as previously described [13], [14]. Twenty-four hours post exposure the mice were sacrificed with CO_2_ asphyxiation. Histological analysis of H&E stained slides were used to determine perivascular vascular inflammation (PVI) using a modified quantification schema [15]. Briefly, the intensity of perivascular inflammation was scored on a scale of 1 to 9. 0, was no inflammation; 1–3, was scant cells but not forming a defined layer; 4–6, one to three layers of cells surrounding the vessel; 7–9, four or greater layers of cells surrounding the vessel. Ten measurements were made from 20-x H&E images per mouse and the slides were coded so that the reviewer did not know the exposure status of the mice. Immunoreactivity assays were performed on paraffin-embedded samples (6 µm), as previously described [16]. Mouse lung sections from control, cigarette smoke and biomass smoke exposed were stained with rabbit polyclonal anti-phospho-ERK, anti-phospho-p38 and anti-phospho-JNK (Cell signaling Technologies, Danvers, MA; #4370, 4511 and 9255 respectively) antibodies. Isotype control rabbit IgG were used as negative controls in each assay. Alexa fluor secondary antibodies (Invitrogen) were utilized to detect rabbit (488 nm) primary antibody targets. The Columbia University Institutional Animal Care and Use Committee approved all animal studies.

### Statistical analyses

Data are expressed as means ± S.E.M. We determined statistical significance by one-way analysis of variance for multiple group analysis using GraphPad Prism Software. Student t-tests (two tailed) were used throughout the study. All data sets are represented as mean +/− standard error.

## Results

### Biomass exposure alters MMP and cytokine secretion and MAPK signaling in SAECs

**Figure 2:**
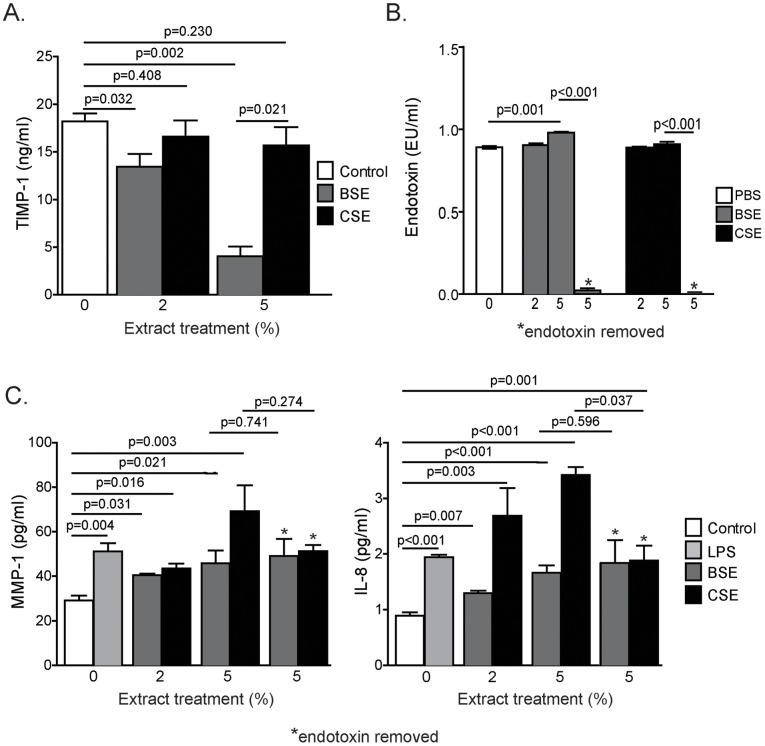
The effect of BSE and CSE on TIMP-1, MMP-1 and IL-8 expression in human SAE cells. (A) TIMP-1 levels were measured from the media of SAE cells treated with 2% or 5% of BSE or CSE for 24 hours via ELISA. N = 6 per group. Data is reported as mean ± standard error of measurement. (B) Endotoxin levels were measured in phosphate buffered saline (PBS) and biomass smoke extract (BSE) and cigarette smoke extract (CSE) before and after endotoxin removal. N = 4 in each group. Data is reported as mean ± standard error of measurement. (C) MMP-1 (left panel) and IL-8 (right panel) levels were measured from the media of SAE cells treated for 24 hours with 2% BSE, CSE or 5% BSE, CSE with and without endotoxin removal and 1 µg/ml LPS for 24 hours via ELISA. N = 6 per group. Data is reported as mean ± standard error of measurement. *Indicates endotoxin removal.

**Figure 3:**
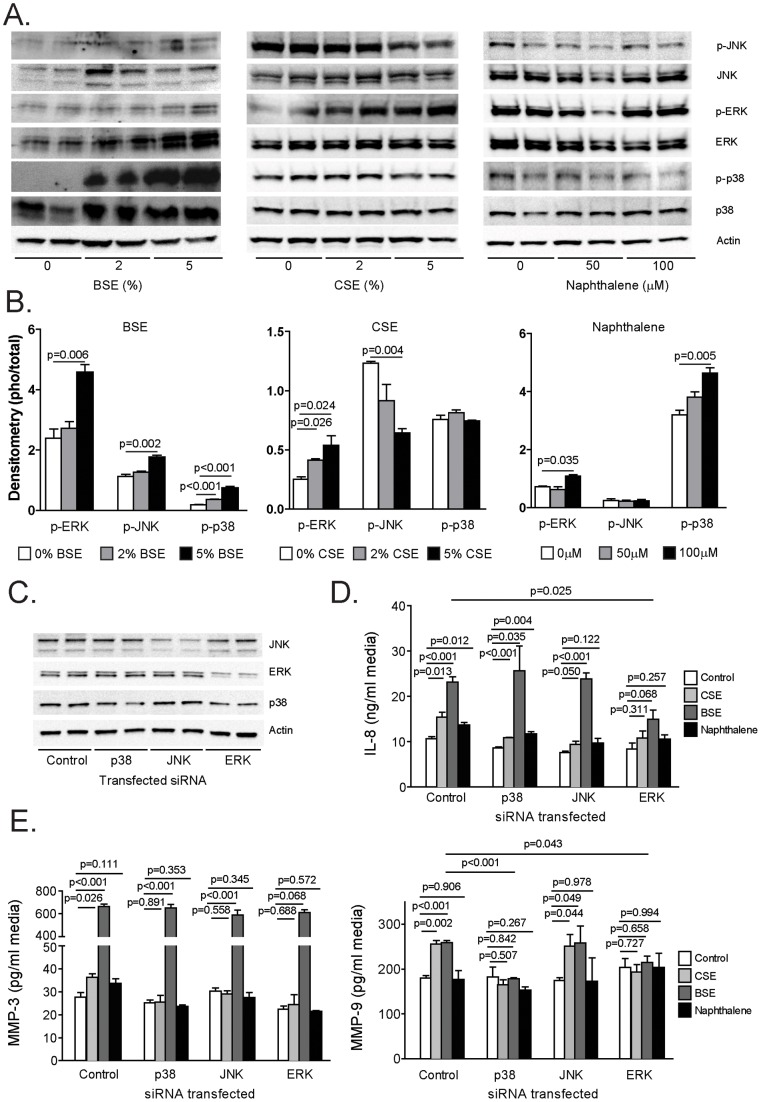
The effect of MAPK signaling on cytokine and protease induction by BSE, CSE and naphthalene in SAE cells. (A) Westerns for active and total JNK, ERK and p38 were conducted on cell lysate protein from SAE cells treated with 2–5% of BSE or CSE or 50–100 μM of naphthalene. Time point was 30 minutes. Actin was used as a loading control. (B) Densitometric analyses were conducted comparing the ratio of active to total levels of ERK, JNK and p38 in the above treated cells. (C) Westerns for total JNK, ERK and p38 were conducted on cells pre-treated with siRNA for each of these MAPK proteins. Actin was used as a loading control. (D) SAE cells were treated with control siRNA or siRNA for p38, JNK or ERK for 6 hours. These SAE cells were then treated for 24 hours with 2% BSE, CSE or 100 μM of naphthalene. IL-8 levels were determined in the media using multiplex analysis. N = 6 per group. Data is reported as mean ± standard error of measurement. (E) MMP-3 (left panel) and MMP-9 (right panel) were measured via multiplex analysis in SAE cells that had been treated with siRNA and then exposed to 2% BSE, CSE or 100 μM of naphthalene for 24 hours. N = 6 per group. Data is reported as mean ± standard error of measurement.

A schema of our *in vitro* model is presented in **Fig. 1**. In contrast to cigarette smoke, BSE caused a dose dependent decrease in TIMP-1 expression in these cells (**Fig. 2A** **)**. High quantities of endotoxin have been reported within households that use dung biomass as a fuel [17] and endotoxin induces both MMP-1 and IL-8 expression [18], [19]. A small but significant increase in endotoxin levels was measured in BSE at a concentration of 5% (**Fig. 2B**). To determine whether the effects of biomass were endotoxin mediated, MMP-1 and IL-8 levels were measured in the BSE and CSE before and after endotoxin removal (**Fig. 2C**)**.** LPS, BSE and CSE treatment all significantly increased MMP-1 (**Fig. 2C, left panel**) and IL-8 (**Fig. 2C, right panel**) protein levels in the media of treated SAECs (dose dependent). Despite eliminating endotoxin, protein levels for MMP-1 and IL-8 remained significantly increased by BSE treatment (**Fig. 2C**). Thus, endotoxin does not account for the induction of MMP-1 and IL-8 in response to biomass in these cells. In contrast, the effect of CSE on IL-8 levels was significantly decreased by the removal of endotoxin. To determine if other components of biomass may account for the effects seen, signaling responses were compared between cells treated with biomass, cigarette smoke or naphthalene. Naphthalene is produced by combustion of organic material, such as tobacco, wood, petroleum and coal, and it is one of the best-characterized components of dung biomass smoke [12]. BSE activated JNK, ERK and p38 while CSE decreased JNK activation, increased ERK and had no effect of p38 (**Figure 3** **, left and center panels**). In comparison, naphthalene had no effect on JNK but stimulated both ERK and p38 (**Figure 3, right panel**). Changes in MAPK signaling were quantified by calculating the ratio of active to total MAPK protein via densitometric analysis (**Figure 3B**). SAECs were then treated with siRNA for p38, JNK or ERK (**Figure 3C**) to determine which MAPK signaling proteins were responsible for the cytokine and protease induction mediated by these stimuli. The loss of ERK expression prevented the induction of IL-8 by all three stimuli (**Figure 3D**). On the other hand, none of the MAPK proteins regulated the expression of MMP-3 (**Figure 3E**). MMP-9 induction, however, was significantly blunted by the loss of p38 or ERK expression in these studies (**Figure 3F**). Of note, biomass exposure at the concentrations reported in this study did not alter cell viability as determined by Alamar blue viability assay. However, viability was affected when the cells were treated with high concentrations of 10% BSE.

### Effects of biomass and cigarette smoke exposure in mice

**Figure 4:**
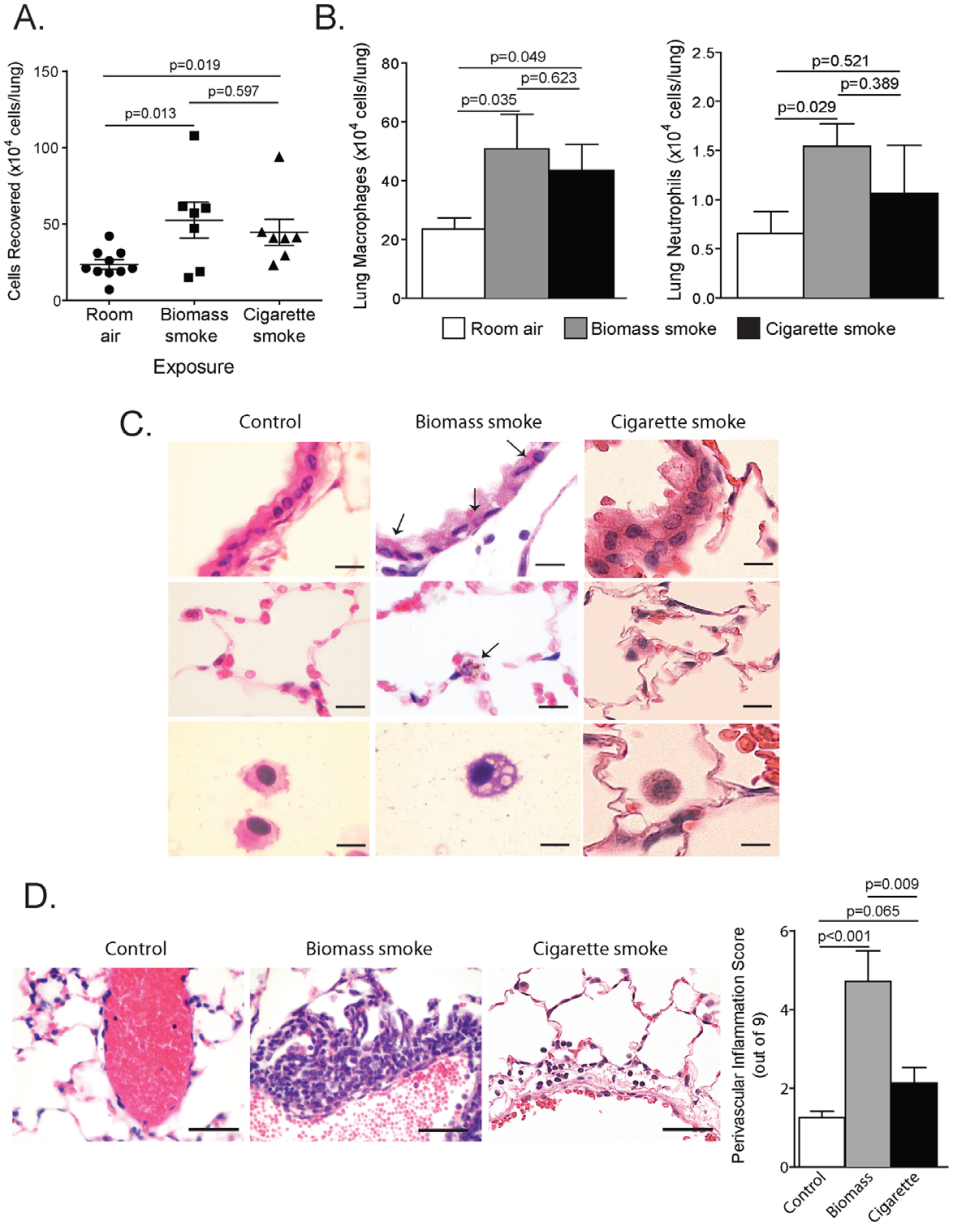
The effect of biomass exposure on lung inflammation, MAPK signaling and protease expression in mice. (A) Lung bronchoalveolar lavage (BAL) cellularity (left panel) was measured in C57BL/6 mice exposed to room air, biomass smoke or cigarette smoke for one week. (B) Quik-diff analysis of the lung lavage cellular pellet was used to calculate the total number of macrophages (top left panel) or neutrophils (top right panel) in the lung lavage. N = 8 in each group. (C) H&E stained lung tissue sections of control (left panels), biomass-exposed (center panels) or cigarette smoke-exposed mice (right panels). In exposed mice, biomass material (see arrows) can be seen within the airway epithelium (top center panel) and alveolar region (middle center panel). The bottom panels demonstrate that macrophages from biomass-exposed mice (bottom center panel) were larger and were full of vacuoles that were not present in the control macrophages (bottom left panel) (scale bar = 20 µM). (D) Biomass-exposed mice (center left panel) had intense, lymphocytic, perivascular inflammation. These changes were not observed in control mice (top left panel) and were less notable in cigarette smoke-exposed mice (bottom left panel) (scale bar = 20 µM). Perivascular inflammation scores were calculated for control, biomass-exposed and cigarette smoke-exposed mice (right panel). N = 8 in each group. Data is expressed as mean ± standard error of measurement.

**Figure 5:**
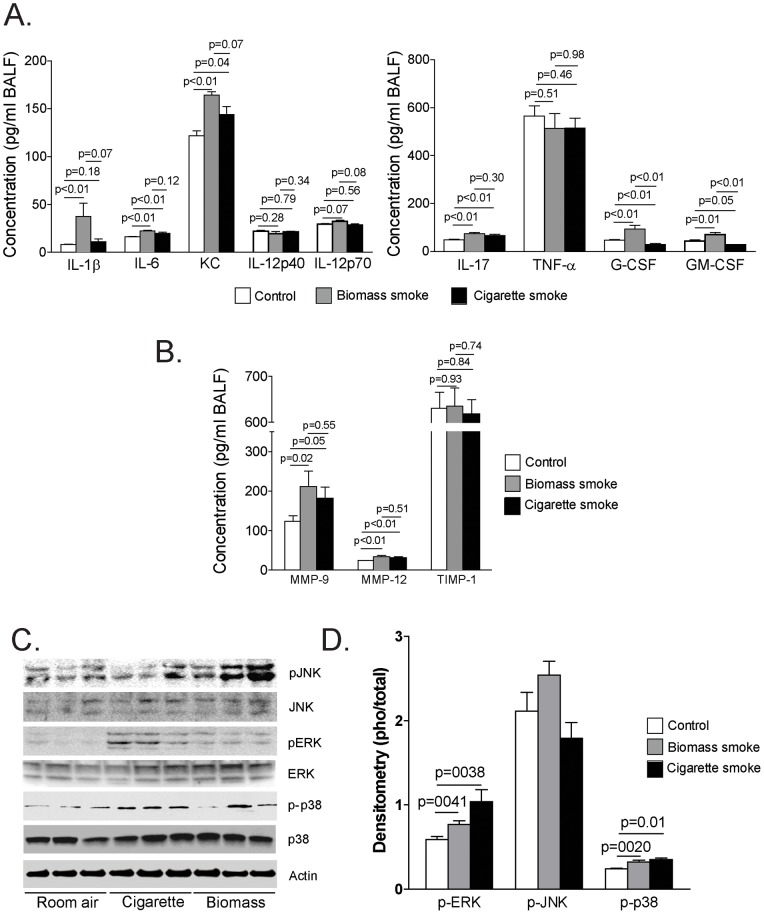
Effects of biomass or cigarette smoke exposure on cell signaling and cytokine and protease levels in mice. (A) Lung lavage (BALF) levels of IL-1β, IL-6, KC, IL-12p40, IL-12p70, IL-17, TNF-α, G-CSF and GM-CSF were measured via multiplex analysis in control mice and mice exposed to one-week of biomass or cigarette smoke. N = 10 in each group. (B) Lung lavage (BALF) levels of MMP-9, MMP-12 and TIMP-1 were measured via multiplex analysis in control mice and mice exposed to one-week of biomass or cigarette smoke. N = 10 in each group. (C) Western blot analysis for active and total p38, ERK and JNK were determined on lung lysate protein from the room air, cigarette smoke or biomass-exposed mice. (D) Densitometric analyses were conducted comparing the ratio of active to total levels of ERK, JNK and p38 in the lungs of the above treated mice.

**Figure 6:**
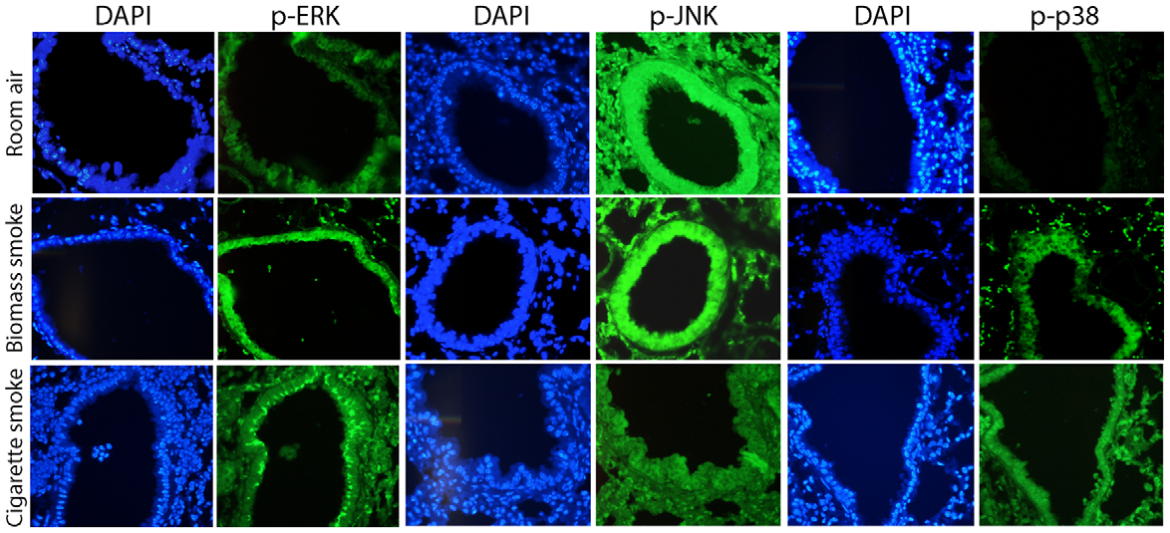
Immunofluorescent analysis of MAPK signaling in the lungs of biomass or smoke exposed mice. Immunofluorescence (FITC-green) for p-ERK, p-JNK and p-p38 was conducted on lung tissue sections from control mice and mice exposed to one week of biomass or cigarette smoke. DAPI staining was used to visualize nuclei.

Mice were exposed to biomass for 30 minutes twice a day for one week and compared to unexposed mice and mice that were exposed to one-week of cigarette smoke as per our standard protocol [14]. Lung lavage cellularity was significantly increased in biomass and cigarette smoke-exposed mice compared to non-exposed controls (530,000±120,000 vs. 180,000±40,000, p<0.02) (**Fig. 4A**). Though the overall particulate matter exposure for the biomass-exposed mice was less (200 mg/m^3^/day for biomass smoke vs. 480 mg/m^3^/day for cigarette smoke), both groups had similar inflammatory responses. Moreover, the overall number of macrophages within the lavage was significantly increased in the biomass and smoke-exposed mice (**Fig. 4B, left panel**). Interestingly, the increase in lavage neutrophils was only significant in the mice exposed to biomass smoke (**Fig. 4B, right panel**). Biomass exposed mice exhibited deposits of carbonaceous material in the lung epithelial cells and macrophages (**Fig. 4C** **, upper center and middle center panels**) and these macrophages contained large vacuoles indicative of an activated state (**Fig. 4C** **, bottom center panel**)**.** These findings were not noted in the other groups of mice. Furthermore, both biomass and cigarette smoke-exposed mice exhibited perivascular lymphocytic inflammation (**Fig. 4D, left panel**). However, the intensity of that inflammation was significantly greater in the biomass-exposed mice (4.7±0.78 for biomass smoke-exposed vs. 2.1±0.40 for cigarette smoke-exposed, p<0.009) (**Fig. 4D, right panel**)**.** To characterize the inflammatory and proteolytic responses produced by these exposures, we measured lung lavage cytokines and proteases within the lung lavage fluid of exposed mice. Both biomass and cigarette smoke increased IL-6, KC and IL-17 (**Fig. 5A**) yet IL-1β, G-CSF and GM-CSF were only increased in the biomass cohort (**Fig. 5A**). In terms of proteases, both MMP-9 and -12 were significantly increased in the biomass and cigarette smoke-exposed mice while TIMP-1 levels were unaffected (**Fig. 5B**). Biomass exposure activated all three MAPK proteins in the lung while cigarette smoke activated only ERK and p38 (**Figs. 5C**). Changes in MAPK signaling were quantified by calculating the ratio of active to total MAPK protein via densitometric analysis (**Fig. 5D**). Using lung tissue sections from these mice, immunofluorescence was conducted staining for the active form of each MAPK protein. Both biomass and cigarette smoke activated ERK within the airways (**Fig. 6**). Though active JNK was seen in the biomass-exposed mice, the mice exposed to cigarette smoke actually had lower levels of JNK activation in their airways (**Fig. 6**). The decrease in JNK in response to cigarette smoke is consistent with what others and we have reported [14], [20]. Lastly, both biomass and cigarette smoke exposure increased p38 activation within the lung airway epithelium (**Fig. 6**).

## Discussion

This study provides a detailed characterization the biologic effects of dung biomass exposure, a fuel source that is utilized by hundreds of millions of people worldwide [12]. Comparative analyses demonstrated that both biomass and cigarette smoke activate pathogenic processes, such as inflammation and protease expression, that are linked to the development of COPD. There is an increasing awareness that indoor air pollution is a significant contributor to the worldwide burden of this disease [21]. Indeed, the use of biomass fuels has been associated with worsened respiratory symptoms, reduced lung function and the development of COPD [22], [23]. Moreover, human studies show that the link between biomass exposure and airway obstruction is strong even after adjusting for other COPD risk factors [24]. The data presented in this work provides a biological rationale for that association as biomass was found to activate mechanisms in the lung that are central to the development of COPD [25]–[27]. Together with emerging data from clinical studies, these findings provide evidence that public health interventions are urgently needed to address this global health threat.

Biomass activated ERK and p38, which are MAPK signaling proteins that play an important role in the development of chronic airway inflammation [28], [29]. Both biomass and cigarette smoke increased protease expression. Biomass, however, induced a greater imbalance between proteases and anti-proteases in human small airway epithelial cells indicating that that this exposure could generate more destruction in the small airways, a critical region in this disease [30]. Another distinction between the two exposures was that biomass was associated with higher levels of G-CSF and GM-CSF in the lung lavage post exposure. This is significant as these cytokines mediate neutrophil influx in this disease [31], [32] and may account for the higher neutrophil levels observed in the biomass exposed mice. In contrast to cigarette smoke, the effect of biomass on IL-8 induction was only partly dependent on endotoxin. Moreover, administration of naphthalene, a major component of dung biomass smoke, did not reproduce the effects seen with biomass smoke extract treatment. siRNA studies demonstrated that specific MAPK proteins mediated the cytokine and protease production in response to these stimuli. Additional unidentified signaling pathways are likely involved in the pathogeneic response as the induction of MMP-3 was completely unaffected by silencing MAPK protein expression. Future studies will need to further address the specific biomass components and signaling processes that are responsible for the damaging effects in the lung.

The activation of the distinct MAPK signaling pathways noted in this study could lead to pathologic changes from biomass smoke that are different from cigarette smoke related lung disease. In support of this, an intense, perivascular lymphocytic inflammation was observed in the lungs of the biomass-exposed mice. This is a finding reported to occur in human COPD [33] that may play a role in the development of pulmonary hypertension [34] but is typically not seen in the mouse model of disease. It is important to note that our exposures were relatively brief. Thus, additional long-term studies will be needed to fully characterize the chronic effects of biomass exposure in this model. Compared to cigarette smoke exposure, lung levels of active JNK were higher in the biomass-exposed mice. Interestingly, studies have demonstrated an association of JNK activation with perivascular inflammation after smoke-inhalation injury [35]. Lymphocytic inflammation leads to the development of pulmonary hypertension [36] and biomass exposure is reported to be a risk factor for cor pulmonale [37]. In fact, the frequency of pulmonary hypertension is higher in female COPD cases due to biomass smoke than in male COPD cases due to tobacco smoke [38]. Together, these findings suggest that dung biomass exposure leads to molecular changes linked to the development of COPD and may induce pathogenic changes that contribute to pulmonary hypertension. This potential link will need to be explored more fully in future studies of biomass exposure.

Macrophages from biomass-exposed mice exhibited prominent vacuoles and carbonaceous material within their cytosol. When activated, macrophages produce high levels of MMP-9 and -12 [39] and the expression of these proteases was increased in the mice exposed to dung biomass smoke. The release of these proteases is known to lead to tissue destruction and emphysema formation [40], [41]. These data indicate that biomass exposure activates macrophages to cause lung-remodeling changes that result in airway obstruction. Unlike cigarette smoke, exposure to biomass down regulated TIMP-1 expression in small airway epithelial cells creating a protease-anti-protease imbalance that is known to be particularly damaging to the lung [25], [42]. The *in vivo* studies however did not replicate the differential response of TIMP-1 to cigarette smoke and biomass. This is likely due to the fact that the *in vivo* analysis reflects the majority of changes from macrophage TIMP-1 obscuring the changes in epithelial cell production. Future studies will need to further evaluate the effect of biomass on the protease/anti-protease balance in the lung.

This study fills a critical gap in the existing literature and provides new insights into the effects of biomass combustion on lung health. Based on our results, we assert that interventions that limit the intensity or duration of this exposure will have a major impact on global public health. Moreover, our research models could be utilized in intervention studies to evaluate the efficacy of improved stove technologies on respiratory health.
